# Study on the pathogenesis of Holmes tremor by multimodal 3D medical imaging: case reports of three patients

**DOI:** 10.1186/s12883-021-02503-2

**Published:** 2021-12-06

**Authors:** Min Shi, Anrong Wang, Yu Fang, Jun Guo, Zhaoying Li, Suoguo Jin, Huan Zhao

**Affiliations:** Department of Neurology, Hospital of Chengdu University of Traditional Chinese Medicine, No. 39 Shi-er-qiao Road, Sichuan Province 610072 Chengdu, P R China

**Keywords:** Holmes tremor, Multimodal 3D medical imaging, Thalamic nuclei, Nerve fiber tract, Case report

## Abstract

**Background:**

We examined for the first time the imaging characteristics of Holmes tremor (HT) through multimodal 3D medical imaging.

**Case presentation:**

Three patients with Holmes tremor who visited the Affiliated Hospital of Chengdu University of TCM from August 2018 to April 2021 were retrospectively investigated to summarize their clinical and imaging data.

**Results:**

Holmes tremor in two of the three patients was caused by hypertensive cerebral hemorrhage and in the third patient induced by hemorrhage due to ruptured brain arteriovenous malformations. HT occurred 1 to 24 months after the primary disease onset and manifested as a tremor in the contralateral limb, mostly in the upper portion. Cranial MRI showed that the lesions involved the thalamus in all three patients. The damaged thalamic nuclei included the ventral anterior nucleus, ventral lateral nucleus and ventromedial lateral nucleus, and the damaged nerve fibers included left thalamocortical tracts in one patient. In the other two patients, the damaged thalamic nuclei included the centromedian and dorsomedial nucleus, and the damaged nerve fibers included left cerebellothalamic and thalamocortical tracts. One patient showed significant improvement after treatment with pramipexole while the other two patients exhibited a poor response, one of whom had no response to the treatment with pramipexole and was only significantly relieved by clonazepam.

**Conclusion:**

We used multimodal 3D medical imaging for the first time to analyze the pathogenesis of HT and found that multiple thalamic nuclei were damaged. The damaged nuclei and nerve fiber tracts of two patients were different from those of the third patient, with different clinical manifestations and therapeutic effects. Therefore, it is speculated that there may be multiple pathogeneses for HT.

## Background

Holmes tremor (HT) is an irregular, low-frequency (< 4.5 Hz) tremor that is predominantly unilateral and that often occurs in the upper limbs with occasional involvement of the lower limbs. The differential etiologies include ischemic or hemorrhagic cerebrovascular disorders, bleeding secondary to vascular malformations, demyelination, and infection. Currently, it is speculated that HT is related to central pathway remodeling [1, 2], but its pathogenesis remains unclear. To help investigate this point, we utilized multimodal 3D imaging of the brain to obtain direct imaging evidence of HT. In this study, three imaging characteristics were investigated to strengthen the clinical understanding of HT.

## Case presentation

### Subjects and methods

#### Subjects

The data from three HT patients who consecutively visited the Department of Neurology, Affiliated Hospital of Chengdu University of TCM from August 2018 to April 2021 were retrospectively analyzed. In particular, their clinical and imaging characteristics were investigated. The diagnostic criteria for HT in this work complied with the Consensus Statement on the Classification of Tremors established by the International Parkinson and Movement Disorder Society in 1998 [3], as were as follows: (1) rest and intention tremors, mostly accompanied by postural tremors; (2) usually lower than 4.5 Hz; (3) mostly occurring in 1 to 24 months after the primary disease onset.

#### Clinical information

Clinical information was collected from the patients, and included gender, age of onset, etiology, delay in HT onset time after primary disease onset, side of tremor (left/right), affected limb (upper/lower limb), presence or absence of hemiplegia, aphasia or paresthesia, involvement of cranial nerves, treatment methods, and outcome.

#### Neurophysiological testing

Electromyography (EMG) tremor analysis was performed in the patients using the Nicolet EDX 6-channel EMG system with 4 pairs of EMG surface electrodes and 2 piezoresistive accelerometers. Recording parameters were as follows: sensitivity: 100 μV/D, specific scanning speed: 100 ms/ D, EMG low frequency band pass filter: 10.0 Hz, high frequency band pass filter: 10.0 kHz; accelerometer low frequency band pass filter: 0.5 Hz, high frequency band pass filter: 30 Hz. The recording electrodes were placed on the muscle belly of the flexor carpi ulnaris and extensor carpi ulnaris on both forearms, the reference electrodes were placed on the corresponding distal tendons, and the 2 accelerometers were placed 2 cm proximal to the third metacarpophalangeal joint on the dorsal side of both hands. Measurements were recorded at several states, including stillness, posture, intention, and the holding of a load of 1000 g. Stillness was defined as the patient sitting on a chair with both forearms resting on the chair armrests, with the wrists naturally hanging down and fully relaxed. Posture was defined as when the patient’s hands were stretched forward flat with the wrists straight. Intention was defined as when the patient slowly repeated the finger-to-nose movement with the bilateral upper limbs. Finally, holding a load of 1000 g was performed with the hands being stretched forward flat while holding a 1000-g sandbag with the wrists straight. The tremor peak frequency, flexor and extensor tremor half-width power, as well as types of agonist and antagonist muscle contractions were recorded.

#### Multimodal 3D medical imaging

The cranial MRI information of the patients was collected, and included T1-weighted images (T1WI), T2-weighted images (T2WI), FLAIR and diffusion weighted imaging (DWI) sequences, and functional MRI and diffusion tensor imaging (DTI) data. The 3D reconstruction and visualization of multimodal 3D medical images were adopted to reconstruct and analyze the damaged brain sites and related nerve conduction bundles. This technique involves thalamus subfield parcellation and subfield-based tractography. Specifically, the thalamus from the T1WI was parcellated into 25 different subfields using FreeSurfer [4] with a probabilistic atlas built from histological data [5]. The T1WI was then linearly registered to the b0 image of the diffusion series. The generated transformation matrix was used to map the parcellated thalamus to the diffusion data space. The diffusion tensors throughout the brain of the subject were calculated using the “dtifit” function in FSL. The deterministic tractography was performed on the calculated tensors using each subfield of the thalamus as seeds.

## Results

### Clinical manifestations and outcome

The clinical characteristics of the three HT patients (1 male and 2 females) are summarized in Table 1. Of these three patients, HT was caused by hypertensive cerebral hemorrhage in two patients (patient #1 and #3), and was induced by hemorrhage due to ruptured brain arteriovenous malformations and ruptured cerebral cavernous malformations in the other patient (patient #2). HT occurred half a month, 2 months, and 12 months after the primary disease onset, respectively. All patients presented with contralateral limb tremors, with upper and lower limb tremors presenting simultaneously in two patients (patient #1 and #2). Two patients (patient #1 and #3) had hemiparesis with grade 5 upper limb strength on the affected side, grade 5 upper limb strength on the healthy side, and grade 4 to 5 lower limb strength. One patient (patient #3) exhibited involvement of the oculomotor nerve nuclei in the midbrain and manifested as left ptosis covering the whole eye. No patient suffered from aphasia or obvious sensory symptoms, hyposmia, limb stiffness, or bradykinesia. The EMG tremor analysis of the three patients revealed that the peak frequency was 2.6 ~ 3.8 Hz for the rest tremor, postural tremor, intention tremor, and tremor when holding a load of 1000 g. The half-width power of the intention tremor was higher than that of the rest tremor. Synchronous contractions of agonists and antagonists were predominant at rest, while alternating contractions were predominant in the postural tremor, intention tremor, and tremor by holding a load. All three patients were treated with pramipexole. Patient #2 and #3 exhibited varying degrees of improvement, but patient #1 did not respond to pramipexole treatment and was only significantly relieved after switching to clonazepam (Table 1).

**Table 1:** Clinical characteristics of three patients with Holmes tremor

Patient	Gender	Age of Onset	Delay in HT Onset Time (Months)	Past Medical History	Etiology	Lesion Location	Side of Tremor	Limb with Tremor	Hemiplegia	Aphasia	Paresthesia	Involvement of Oculomotor Nucleus	Treatment Method, Remission Degree and Duration
Case 1	Female	73	12	Hypertension for 2+ years	HICH	Left thalamus	Right	Upper + lower limbs	Yes	None	None	None	The symptoms disappeared 1 month after treatment with 2 mg clonazepam Tablets (qd po).
Case 2	Female	23	2	None	AVM	Left thalamus	Right	Upper + lower limbs	None	None	None	None	pramipexole 0.375 mg/d, 20%, 1 month
Case 3	Male	71	0.5	Hypertension 2 years	HICH	Right thalamus	Left	Upper limbs	Yes	None	None	Yes	pramipexole 0.375 mg/d, 80%, 1 month

*Note*: *HICH* hypertensive cerebral hemorrhage, *AVM* arteriovenous malformation

### Cranial imaging

**Fig. 1:**
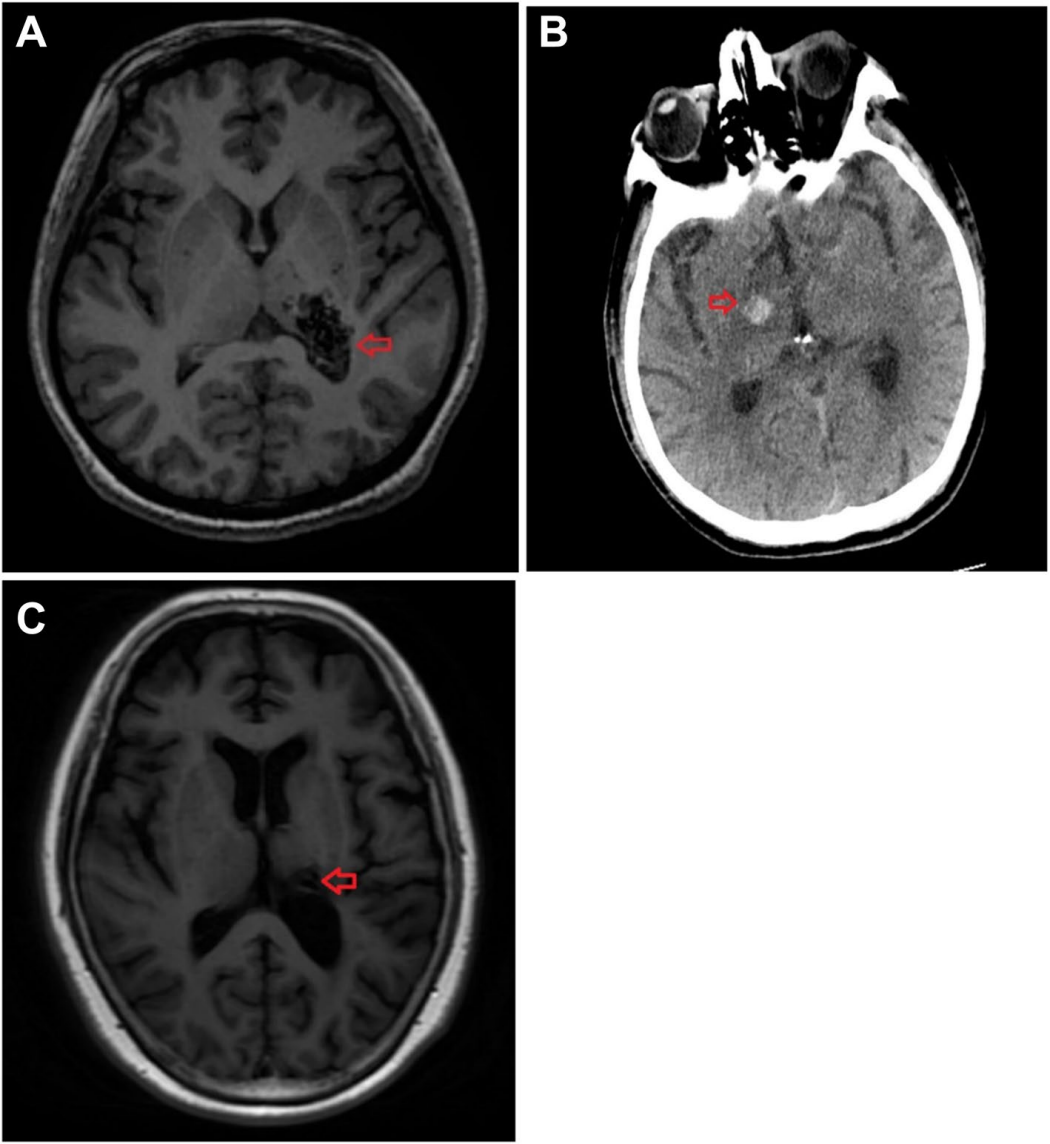
Cranial imaging of the three patients. **A** MRI of patient #1; **B** CT of patient #3; **C** MRI of patient #2

The brain lesions involved the thalamus in all three patients. The MRI of patient #1 revealed old hemorrhagic encephalomalacia lesions, which manifested as patchy long T1 and long T2 signal shadows in the left thalamus and the left posterior limb of the internal capsule (Fig. 1A). The thalamus was involved with hemorrhage in patient #3 and the CT findings showed a high-density shadow in the junction area between the right thalamus and the right anterior limb of the internal capsule, suggesting a new hemorrhagic focus (Fig. 1B). MRI suggested old hemorrhagic softening lesions in patient #2, which manifested as patchy long T1 and T2 signal shadows in the left thalamus (Fig. 1C).

### Multimodal 3D medical imaging

**Fig. 2:**
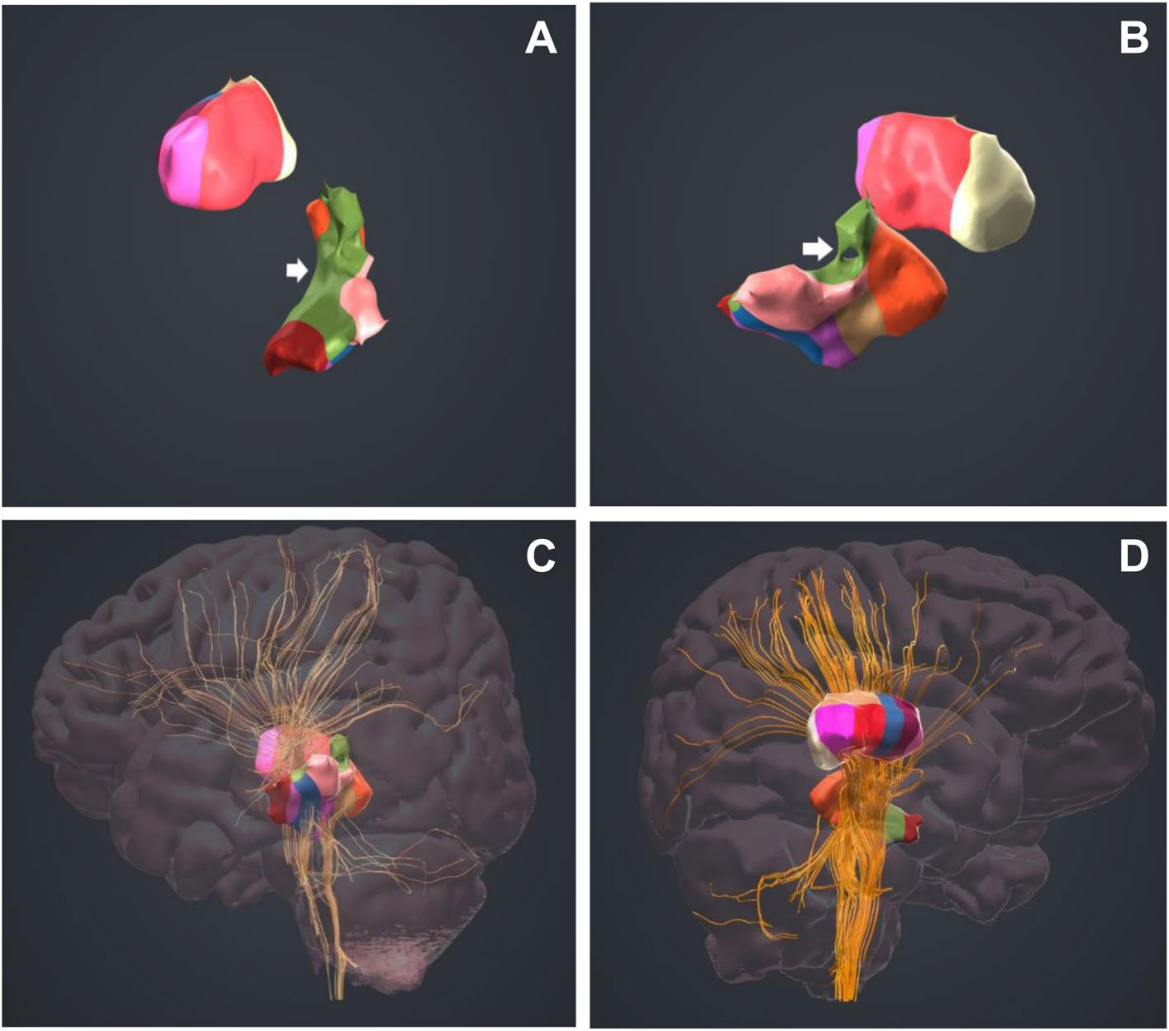
3D reconstruction and visualization of thalamic nuclei and nerve fiber tracts. **A** and **B** show the bilateral thalamus with the white arrows indicating the damaged thalamic nuclei. **C** shows the thalamocerebellar tracts and thalamocortical tracts of the damaged thalamic nuclei, which are significantly reduced compared with those of the undamaged contralateral thalamic nuclei in (**D**)

With the 3D reconstruction and visualization of medical images, the thalamus can be subdivided into the anterior, ventral anterior, ventral lateral, ventromedial lateral, ventral posterolateral, ventral posteromedial, dorsomedial, lateral dorsal, centromedian, pulvinar and posterolateral nuclei. Furthermore, the nerve fibers of thalamic nuclei can be separated one by one to evaluate the damaged nerve fiber tracts. The damaged thalamic nuclei and nerve fiber tracts of the three patients are shown in Table 2, Fig. 2, and Video 1.

**Table 2:** Damaged thalamic nuclei and nerve fiber tracts in three patients with Holmes tremor

Patient	Damaged Sites	Damaged Thalamic Nuclei	Damaged Nerve Fiber Tracts
Case 1	Thalamus, posterior limb of the internal capsule	Pulvinar, centromedian nucleus, dorsomedial nucleus	Left cerebellothalamic tract, thalamocortical tract
Case 2	Thalamus	Dorsomedial nucleus, centromedian nucleus	Left cerebellothalamic tract, thalamocortical tract
Case 3	Thalamus	Ventral anterior nucleus, ventral lateral nucleus, ventromedial lateral nucleus	Right thalamocortical tract

## Discussion

HT is a symptomatic and low-frequency (< 4.5 Hz) tremor that predominantly affects the proximal limbs, mainly the upper limbs and occasionally the lower limbs [1, 2]. A delay of 4 weeks to 2 years is commonly observed between the onset of the lesion and the occurrence of tremor, a process that may be related to nerve remodeling [3]. At present, the pathogenesis of HT is generally believed to be related to the nigrostriatal pathway and/or the cerebello-thalamo-cortical pathway [6]. However, there is no direct imaging evidence to prove the pathogenesis of this disease. To help address this gap, we employed multimodal 3D imaging techniques to elucidate the thalamic nuclei and nerve fibers that are implicated in HT. By analyzing the imaging data, whole-brain DTI, and multimodal 3D medical imaging data of three HT patients, we found that thalamic nuclei damage in one patient (patient #3) involved the ventral anterior, ventral lateral, and ventromedial lateral nuclei, which are parts of the subthalamus. The subthalamic nucleus functionally belongs to the basal ganglia and is closely related to the globus pallidus [7–10]. Damage to this nucleus would cause the well-known hemiballismus. The thalamic nuclei damage in the other two patients involved the centromedian and dorsomedial nuclei, and the nerve fiber damage involved the left cerebellothalamic and thalamocortical tracts. Based on the relevant literature, the centromedian nucleus is the largest nucleus among the intralaminar nuclei of the thalamus and provides the primary nonspecific projection of the thalamus. The afferent impulses of the intralaminar nuclei originate from the ascending fibers of the brainstem reticular formation, the emboliform nuclei of the cerebellum, the medial globus pallidus and other thalamic nuclei [11]. Instead of projecting to the cerebral cortex, the centromedian nucleus projects to the caudate nucleus, putamen, and globus pallidus, and may also diffuse to all thalamic nuclei, which in turn transmit impulses to secondary centers in the cerebral cortex [12]. In summary, the centromedian nucleus is connected not only to the cerebellum, but also to the cerebral cortex. Therefore, we speculate that the centromedian nucleus is the main functional thalamic nucleus in the cerebello-thalamo-cortical pathway. Furthermore, both patients who presented with simultaneous involvement of the upper and lower limbs exhibited poor or even no effect on symptom relief after oral pramipexole administration. In contrast, the other patient with only upper limb involvement responded favorably to pramipexole treatment. We speculate that the pathogenesis of HT in these 2 patients may be different from that in the other patient, and that there may be multiple pathogeneses for HT.

## Data Availability

All data generated or analyzed during this study are included in this published article.

## Abbreviations

HT: Holmes tremor
TCM: Traditional Chinese Medicine
HICH: Hypertensive Cerebral Hemorrhage
AVM: Brain Arteriovenous Malformation
DTI: Diffusion Tensor Imaging

## References

[1] Samie MR, Selhorst JB, Koller WC (1990). Post-traumatic midbrain tremors. Neurology.

[2] Liu ZQ, Wan ZR, Jia XT, Yang XZ, Fang XX, Zhang ZY (2019). Clinical features and short-term prognosis of Holmes' tremor. Zhonghua Yi Xue Za Zhi.

[3] Gajos A, Bogucki A, Schinwelski M, Sołtan W, Rudzińska M, Budrewicz S (2010). The clinical and neuroimaging studies in Holmes tremor. Acta Neurol Scand.

[4] Fischl B (2012). FreeSurfer Neuroimage.

[5] Iglesias JE, Insausti R, Lerma-Usabiaga G, Bocchetta M, Van Leemput K, Greve DN (2018). A probabilistic atlas of the human thalamic nuclei combining ex vivo MRI and histology. Neuroimage.

[6] Deuschl G, Bain P, Brin M (1998). Consensus statement of the Movement Disorder Society on Tremor. Ad Hoc Scientific Committee. Mov Disord.

[7] Kilbane C, Ramirez-Zamora A, Ryapolova-Webb E, Qasim S, Glass GA, Starr PA (2015). Pallidal stimulation for Holmes tremor: clinical outcomes and single-unit recordings in 4 cases. J Neurosurg.

[8] Alqwaifly M (2016). Treatment responsive Holmes tremor: case report and literature review. Int J Health Sci (Qassim).

[9] Krack P, Deuschl G, Kaps M, Warnke P, Schneider S, Traupe H (1994). Delayed onset of “rubral tremor” 23 years after brainstem trauma. Mov Disord.

[10] Gajos A, Budrewicz S, Koszewicz M, Bieńkiewicz M, Dąbrowski J, Kuśmierek J (2017). Is nigrostriatal dopaminergic deficit necessary for Holmes tremor to develop? The DaTSCAN and IBZM SPECT study. J Neural Transm (Vienna).

[11] Duus P (2003). Neurologisch-topische Diagnostik: Anatomie-Funktion-Klinik.

[12] Joutsa J, Shih LC, Fox MD (2019). Mapping holmes tremor circuit using the human brain connectome. Ann Neurol.

